# Identifying peripersonal space boundaries in newborns

**DOI:** 10.1038/s41598-019-45084-4

**Published:** 2019-06-28

**Authors:** Giulia Orioli, Alessandro Santoni, Danica Dragovic, Teresa Farroni

**Affiliations:** 1 School of Psychology, University of Birmingham, Birmingham, United Kingdom; 2 Department of Developmental Psychology and Socialization, University of Padova, Padova, Italy; 3 Paediatric Unit, Hospital of Monfalcone, Monfalcone, GO Italy

**Keywords:** Neuroscience, Psychology

## Abstract

Peripersonal space immediately surrounds the body and can be represented in the brain as a multisensory and sensorimotor interface mediating physical and social interactions between body and environment. Very little consideration has been given to the ontogeny of peripersonal spatial representations in early postnatal life, despite the crucial roles of peripersonal space and its adaptive relevance as the space where infants’ earliest interactions take place. Here, we investigated whether peripersonal space could be considered a delimited portion of space with defined boundaries soon after birth. Our findings showed for the first time that newborns’ saccadic reaction times to a tactile stimulus simultaneous to sounds with different intensities changed based on the sound intensity. In particular, they were significantly faster when the sound was lounder than a critical intensity, in a pattern that closely resembled that showed by adults. Therefore, provided that sound intensity on its own can cue newborns’ sound distance perception, we speculate that this critical distance could be considered the boundary of newborns’ rudimentary peripersonal space. Altogether, our findings suggest that soon after birth peripersonal space may be already considered as a bounded portion of space, perhaps instrumental to drive newborns’ attention towards events and people within it.

## Introduction

Peripersonal space is the portion of space that immediately surrounds the body. It has been suggested that it can be represented as a multisensory and sensorimotor interface mediating both social and physical interactions between the body and the environment^1–4^. In fact, peripersonal spatial representations are thought to have the dual function^1,2^ of supporting goal-directed actions^5^ and enabling us to defend ourselves from imminent threats^6,7^.

Despite the special status of peripersonal space in the human brain^8,9^, which is reflected by the extensive research dedicated to the understanding of its functions^2,5,7,10^, plasticity^5,11–19^ and neural underpinnings^20–34^, and the crucial roles that peripersonal spatial representations play^2^, very little consideration has been given to the ontogeny of peripersonal space representations in early postnatal life. Investigating the representation of peripersonal space in infancy and its development throughout childhood is extremely important, especially in light of the adaptive relevance of peripersonal space and the events taking place in it. In the specific case of newborns, peripersonal space is the portion of space where their earliest interactions with the extrauterine environment take place. These early interactions are invested of particular importance as they hold a central role in shaping newborns’ later development and learning processes. Therefore, it would be reasonable to hypothesize that already during the earliest stages of postnatal life newborns might show a predisposition to pay more attention and react more quickly to the events taking place within peripersonal space, as potential predictors of an upcoming interaction^3,35,36^. Based on this, we wanted to investigate whether soon after birth human newborns show a rudimentary representation of peripersonal space, through the linking of exteroceptive and somatosensory cues. Demonstrating the existence prior to significant postnatal experience of rudimentary associations between exteroceptive signals near the body and tactile stimulation on the body would pave the way to a discussion on whether these associations should be considered to a certain extent innate or rather whether they develop during prenatal life, thanks to multisensory experiences *in utero*.

Recently, a series of studies in adult populations investigated whether peripersonal space could be measured and its boundaries identified^16,37–40^. In particular, Canzoneri *et al*.^37^ implemented a dynamic audio-tactile integration task capable of identifying the boundaries of peripersonal space and of determining its dimensions in an ecologically valid situation. Specifically, the authors measured the participants’ reaction times (RTs) to a tactile stimulus delivered to their hand while an auditory stimulus simulated the motion of a sound source either towards the same hand or away from it. The tactile stimulation was delivered at a number of different delays from the onset of the auditory stimulus, hence occurring when the sound source was perceived at different distances from the hand. The participants were required to respond to the tactile stimulation verbally and as rapidly as possible, trying to ignore the concurrent auditory stimulus. The authors showed that the RTs to the tactile stimulus were speeded up by the presence of a simultaneous sound if this was perceived within a limited distance from the hand, supposedly because of the extremely efficient integration of audio-tactile stimuli that take place within the same spatial representation^37^. They suggested that the critical distance at which the sound source is perceived when the RTs are first speeded up should be considered as the estimated boundary of the representation of peripersonal space around the hand^37^. This approach was also used to investigate how peripersonal space boundaries can be modulated by the participant’s interactions with others in the environment^38^, by the semantic content of the stimuli taking place within it^39^ and by self-directed actions, like walking^16^.

Using an approach similar to that proposed by Canzoneri *et al*.^37^, the present set of studies investigated whether peripersonal space could be considered as a delimited portion of space with identifiable boundaries already during the earliest stages of postnatal life. To this aim, we adapted the task implemented by Canzoneri *et al*.^37^ in order to be able to use it with newborns. In our adaptation, we recorded newborns’ RTs measuring their saccadic latency (sRTs) to two visual targets presented on the screen immediately after the audio-tactile stimulation terminated. The visual targets should not be considered as the stimuli to which we are measuring newborns’ responses, but only as the means to measure said responses. Previous research proved that newborns and young infants show rapid, reflexive eye movements towards easily discriminable stimuli appearing in their temporal hemifield, likely driven by a subcortical mechanism^41^. The subcortical and reflexive nature of these eye movements suggests that they can be considered automatic rather than voluntary orienting responses. Due to their automaticity, the speed of these responses is likely to be influenced by the general state of alertness of the newborns. If newborns had a rudimentary representation of peripersonal space, their alertness state could be in turn modulated by the perception of an audio-tactile stimulus presented close to the body immediately before the visual target. Measuring the saccadic RTs to the visual targets rather than directly to the tactile stimuli would provide an indirect measure of how newborns’ reaction times to the tactile stimulation were affected by the sound but, at the same time, it was the most suitable way to obtain a RTs measure from newborns. In particular, we expected the saccadic RTs to the visual targets to be slightly longer than if it had been possible to measure the RTs to the tactile stimuli themselves. However, we expected this delay to be consistent across the different intensities at which the auditory stimulus was perceived. The reflexive nature of newborns’ visual orienting behaviours^41^ supported our choice to measure saccadic RTs to a visual target ensuring that newborns would orient to said target immediately upon perceiving it. Based on the necessary decision of measuring newborns’ sRTs to visual targets following the audio-tactile stimuli, we decided to use static – rather than dynamic – auditory stimuli, as this would allow us to present the visual targets immediately after the audio-tactile stimulation ended. Sounds with several different intensities were presented: we decided to modulate intensity based on the fact that it is the sound feature that better accounts for sound location in depth^42^ and is also the feature that Canzoneri *et al*.^37^ changed in their task to convey the impression of the sound approaching or receding. We decided to use samples of sinusoidal waveforms instead of samples of pink noise, based on previous findings showing that sounds seem to facilitate both multisensory matching and the detection of changes in intensity compared to noise^43,44^. Furthermore, upon exposing a number of newborns to samples of both pure tones and noise while setting up the study, we noticed that they showed signs of fussiness in response to the noise, while they seemed to be calmer and more attentive when a tone was presented. Finally, we delivered the tactile stimulation by gently stroking the newborns’ forehead using a paintbrush: we choose the forehead to avoid the risk of priming newborns’ saccadic responses to either side of the space by applying a lateralized touch (e.g. on one cheek) that could orient their attention to one or the other side of the screen.

We hypothesized that if peripersonal spatial representations can be considered as bounded already soon after birth, then human newborns will show a pattern of reaction times similar to that shown by adults^37^. In particular, we hypothesized that their reaction times to a touch will be faster when said touch is presented simultaneously to a sound presented with higher vs lower intensity. On the contrary, if peripersonal spatial representations cannot be considered as bounded soon after birth, we expect that newborns’ reaction times will decrease continuously as a function of the sound intensity, or possibly will not change at all.

## Results

First, we ran a pilot study with a small sample of newborns (N = 8) to verify whether our adaption of Canzoneri *et al*.’s^37^ task could be used to capture newborns’ peripersonal space boundaries. Based on the results of the pilot study, we then ran a full experiment (Study 1), involving a larger number of participants (N = 31) and including a control condition in which only auditory, but not tactile, stimuli were used. This condition was included so that if the response pattern identified in the pilot study was replicated in the audio-tactile condition, but not in the auditory only condition, it could be inferred that said pattern should be specifically attributed to the audio-tactile stimulation, ruling out the possibility that it could be driven solely by the perceived intensity of the sound on its own.

### Pilot study

Valid saccadic reaction times (sRTs) were collected for, on average, 50% of the trials attended by the newborns in the final sample. The exact number of valid trials per participant and per condition is summarized in Table 1.

**Table 1:** The table summarises the exact number of valid trials per participant and per condition in both the Pilot Study and Study 1 (in the ID column, “P” refers to Pilot, “SAT” to Study 1 Audio-Tactile group and “SA” to Study 1 Auditory group). If only one valid trial was retained in a specific sound condition, the RT value for that participant and that condition was disregarded.

Study		ID	Sound 0	Sound 1	Sound 2	Sound 3	Sound 5
**Pilot Study**		P_1	n/a	4	n/a	2	3
		P_2	n/a	3	n/a	4	5
		P_3	n/a	7	n/a	6	4
		P_4	n/a	2	n/a	7	3
		P_5	n/a	5	n/a	5	3
		P_6	n/a	4	n/a	4	7
		P_7	n/a	5	n/a	7	5
		P_8	n/a	5	n/a	4	4
**Study 1**	Audio-Tactile Group	SAT_1	1	6	3	4	3
		SAT_2	4	0	2	2	3
		SAT_3	3	2	3	3	3
		SAT_4	2	4	0	3	3
		SAT_5	4	6	2	6	4
		SAT_6	2	4	2	3	4
		SAT_7	4	3	4	4	1
		SAT_8	2	5	2	4	3
		SAT_9	2	4	3	3	2
		SAT_10	3	5	4	3	3
		SAT_11	3	4	3	5	3
		SAT_12	0	4	2	4	3
		SAT_13	3	2	4	3	4
		SAT_14	3	2	4	2	4
		SAT_15	3	6	3	4	5
		SAT_16	3	1	2	3	4
	Auditory Group	SA_1	4	3	3	4	4
		SA_2	2	4	2	4	3
		SA_3	2	2	0	4	2
		SA_4	1	3	3	3	4
		SA_5	3	4	2	3	3
		SA_6	2	3	3	4	4
		SA_7	3	2	2	2	3
		SA_8	1	2	3	4	4
		SA_9	5	3	3	4	5
		SA_10	3	6	3	2	2
		SA_11	2	2	2	3	1
		SA_12	3	1	3	6	6
		SA_13	1	3	2	2	4
		SA_14	2	0	3	2	2
		SA_15	3	2	4	2	1

**Figure 1:**
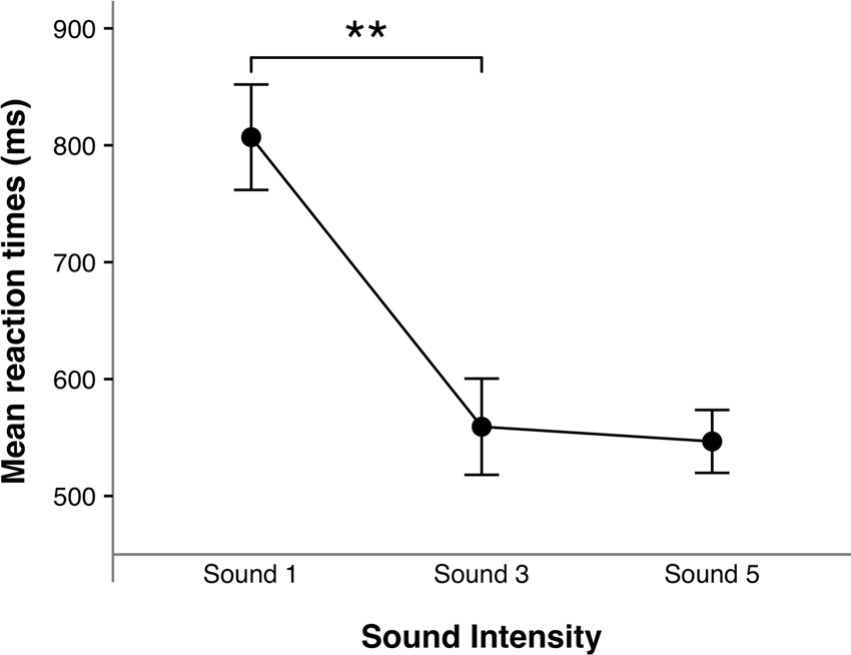
Saccadic reaction times in the Pilot Study. Mean sRTs (and *SE*) in response to the visual targets immediately following the audio-tactile stimulation, as function of the intensity of the sound. Significant comparisons are indicated (**p < 0.01).

We used a mixed-effects model^45^ to investigate the effect of the intensity of the sound on the sRTs to the visual stimuli immediately following the termination of the audio-tactile stimulation. This choice was determined by the possibility given by mixed-effects models to take into account random-effect factors, whose levels are randomly drawn from a population. Two mixed-effects models were tested, one including only Participants as a random factor *(m1)* and one including Sound Intensity condition as a fixed factor and Participants as a random factor *(m*2*)*. Likelihood ratio tests of the full model *(m*2*)* against *m1* showed that the Sound Intensity affected the individual sRTs, *χ*^2^(2) = 17.964, *p* < 0.001. To follow up, we directly compared the sRTs between Sounds 1 and 3 and between Sounds 3 and 5 (the differences between the sRTs in the pairs of conditions were normally distributed - Kolmogorov-Smirnov test: Sounds 1 and 3, *D* = 0.179, *p* = 0.924; Sounds 3 and 5, *D* = 0.176, *p* = 0.930). Planned paired comparisons (Fig. 1) showed that newborns’ sRTs were significantly faster after the sound was perceived at Sound 3 (*M* = 559.25 ms, *SE* = 41.17 ms) vs Sound 1 (*M* = 806.89 ms, *SE* = 45.09 ms), *t*(7) = 3.696, *p* = 0.008, *d*_*z*_ = 1.307, but not at Sound 5 (*M* = 546.67 ms, *SE* = 26.89 ms) vs Sound 3, *t*(7) = 0.291, *p* = 0.779, *d*_*z*_ = 0.103 (in order to correct for multiple comparisons, $\alpha$ = 0.025).

We also wanted to verify if the number and latency of orienting responses to the visual target on the left vs the right sides of the screen were comparable. To do so, we ran two paired planned comparisons per each presented sound, one on the number of orienting responses and one of their latency. None of the comparisons reached significance [all *t* < 1.37, all *p* > 0.21], ruling out the possibility of an effect of the presentation side of the target on newborns’ responses.

### Study 1

The pilot study showed a modulation of newborns’ sRTs following an audio-tactile stimulation. Specifically, the sRTs were significantly speeded up when Sound 3 was presented compared to when Sound 1 was presented, while the sRTs between Sounds 3 and 5 were not significantly different (Fig. 1).

In light of this result, we wanted to further explore newborns’ responses in this task, in particular investigating their sRTs to visual targets appearing immediately after an audio-tactile stimulation in which the auditory stimuli were presented at a larger number of different intensities. We were specifically interested in two additional intensities: one intermediate between Sounds 1 and 3, i.e., where the sRTs were first significantly speeded up in the pilot study (Sound 2), and the second softer than Sound 1 (Sound 0). The reason for adding the latter position lies in the fact that Canzoneri *et al*.^37^ showed that adults’ RTs in response to the touch did not significantly differ from each other when the sound was perceived at any of the perceived sound distances before or after the critical distance, and we wanted to verify whether the same was true also for newborns.

We also wanted to control whether the effect found in the pilot study was specifically related to the simultaneous audio-tactile stimulation rather than being due to the auditory stimulation on its own. In other words, we wanted to rule out the possibility that sRTs could have changed simply as a function of the intensity of the sound, irrespective of the touch. In fact, it might be suggested that louder sounds could on their own modulate newborns’ alertness state, irrespective of any supposed peripersonal spatial representation. We addressed this by testing a second group of newborns that experienced only unimodal auditory (but not tactile) stimulation.

In Study 1, valid sRTs were collected for, on average, 52% of the trials that the newborns in the final sample attended (see Table 1 for details on the individual number of valid trials per condition). There were no substantial differences between the number of trials attended by the newborns allocated to the two different groups (Audio-tactile stimulation group: 55%; Auditory stimulation group: 49%). The valid sRTs recorded per Sound Intensity condition from the newborns in both groups are described in Table 2.

**Table 2:** The table shows newborns’ average newborns’ saccadic reaction times (average sRTs) to the visual target appearing immediately after the audio-tactile stimulation ceased, their standard deviations (*SD*) and standard errors (*SE*), per Sound Intensity condition.

Group	Sound Intensity condition	average sRTs	*SD*	*SE*
Audio-tactile stimulation	Sound 0	804.05	180.54	48.25
Audio-tactile stimulation	Sound 1	805.83	117.02	31.27
Audio-tactile stimulation	Sound 2	602.46	129.99	33.56
Audio-tactile stimulation	Sound 3	597.12	181.80	45.45
Audio-tactile stimulation	Sound 5	556.71	150.28	38.80
Auditory stimulation	Sound 0	694.94	174.80	50.46
Auditory stimulation	Sound 1	687.82	192.19	53.30
Auditory stimulation	Sound 2	671.43	191.33	51.13
Auditory stimulation	Sound 3	659.11	142.83	36.88
Auditory stimulation	Sound 5	649.33	224.05	62.14

**Figure 2:**
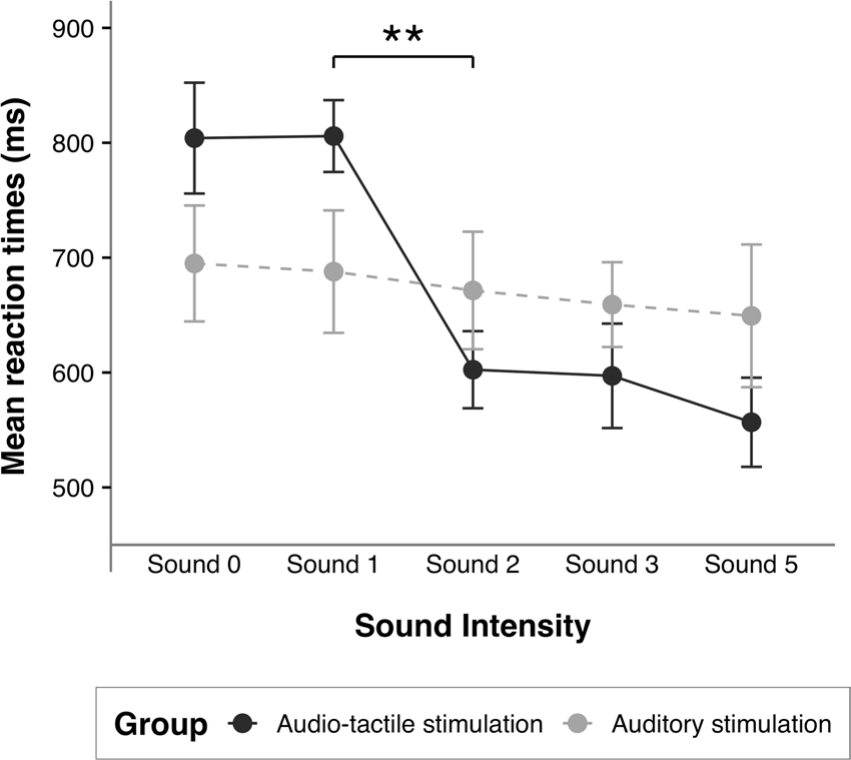
Saccadic reaction times in Study 1. Mean sRTs (and *SE*) in response to the visual targets immediately following the audio-tactile (Audio-tactile stimulation group) or auditory (Auditory stimulation group) stimulation, as function of the intensity of the sound. Significant comparisons are indicated (**p < 0.001).

We used a mixed-effects model^45^ to investigate the effect of the modality of stimulation (audio-tactile vs auditory) and of the intensity of the sound on the sRTs to the visual stimuli immediately following the termination of the audio-tactile stimulation. This choice was determined by the possibility given by mixed-effects models to take into account random-effect factors and to control for uneven numbers of observations. Three mixed-effects models were tested: the first *(m1)* included Stimulation as a fixed factor and Participants as a random factor; the second *(m*2*)* included also Sound Intensity condition as a fixed factor; the third *(m3)* added to *m*2 the interaction between Stimulation and Sound Intensity condition. Likelihood ratio tests showed that the full model *(m3)* was the best in predicting the collected data and that the Interaction between Stimulation and Sound Intensity condition affected the individual sRTs, *χ*^*2*^(4) = 10.391, *p* = 0.034. We therefore tested two separate models per each stimulation group, one including only Participants as a random factor *(m4)* and one including Sound Intensity condition as a fixed factor and Participants as a random factor *(m5)*. In the Audio-tactile stimulation group, likelihood ratio tests of the full model *(m5)* against *m4* showed that Sound Intensity affected the individual sRTs, *χ*^*2*^(4) = 28.654, *p* < 0.001. On the contrary, in the Auditory stimulation group the full model did not explain the collected data better than the model including Participants only *(m4)*, *χ*^*2*^(4) = 0.404, *p* = 0.982. Planned paired comparisons on the sRTs collected from the Audio-tactile stimulation group (Fig. 2) showed a significant difference in newborns’ sRTs only between Sounds 1 and 2, *t*(12) = 4.506, *p* < 0.001, *d*_*z*_ = 1.249 (in order to correct for multiple comparisons, $\alpha$ = 0.0125; all the other comparisons were not significant, see Table 3 for the complete results).

**Table 3:** The table summarises the results of the comparisons between Sound Intensity conditions in the Audio-tactile stimulation group, including Kolmogorov-Smirnov tests (*D*) for testing the normality of the distribution of the differences between pairs of conditions, and paired planned comparisons (*t*) between subsequent Sound Intensity conditions (0 and 1, 1 and 2, 2 and 3, 3 and 5).

Comparisons between Sound Intensity conditions	*D value*	*p value*	*dfs*	*t value*	*p value*	*d*_*z*_
Sounds 0–1	0.163	0.856	11	−0.530	0.607	0.153
Sounds 1–2	0.196	0.629	12	4.506	<0.001	1.249
Sounds 2–3	0.123	0.978	14	0.228	0.823	0.059
Sounds 3–5	0.099	0.995	14	0.623	0.543	0.161

The newborns who participated in the study were randomly assigned to either the multisensory or the unisensory group. This, together with the difficulty of planning when to test newborn participants given their very limited awake time, resulted in a 24-hours difference between the mean ages of the participants included in the two final samples. In order to investigate the possibility that differences in age at the time of testing had an effect on the responses collected, we compared *m3* to a new model *(m6)* including age and its interaction with the other factors. Likelihood ratio tests showed that the full model did not explain the collected data better than *m3, χ*^*2*^(10) = 8.265, *p* = 0.603, ruling out the possibility that the age of the participants had an effect on the data collected. Additionally, to further rule out this possibility, we also correlated the age of participants and their sRTs to each of the sounds presented: this analyses did not reveal any significant correlations between age and sRTs, in neither group [all *R* comprised between −0.33 and 0.36, all *p* > 0.199].

Finally, we verified whether the number and latency of orienting responses to the visual target on the left vs the right sides of the screen were comparable. To this aim, for both groups we ran two paired planned comparisons per each presented sound, one on the number of orienting responses and one of their latency. None of the comparisons reached significance [all *t* < 1.83, all *p* > 0.10], ruling out the possibility of an effect of the presentation side of the target on newborns’ responses.

## Discussion

In the past two decades, a great number of studies shed light on the characteristics of peripersonal space in adults, including the measurement of its dimensions and the definition of its boundaries^2,5,7,10–34,37–40^. Peripersonal space boundaries are defined by a spatially and temporally delimited multisensory facilitation of tactile processing, which is thought to be driven by the importance of nearby and approaching stimuli in predicting an impending interaction between the body and an external stimulus^3,35,36^. However, to our best knowledge, no studies explored if newborns and young infants show any rudimentary peripersonal spatial representation and, should this be the case, if these representations are characterized by identifiable boundaries during development. This topic is of particular interest as peripersonal space is the portion of space where newborns and infants’ earliest interaction with the outside world will take place and especially because these interactions are likely to influence infants’ later development and learning. Another reason why the investigation of peripersonal spatial representations in infancy may be invested of a great theoretical importance is associated to peripersonal space relation with inferential and predictive mechanisms. One of the most relevant and recent theoretical developments on peripersonal space conceptualisation highlights its role in the inferential processes linking events taking place near the body and their impending tactile interactions with the body^3,35,36^. It has been suggested that predictive mechanisms may drive tactile processing when an exteroceptive stimulus is perceived near the body, as the same stimulus could anticipate an impending contact. These predictive mechanisms, by enhancing tactile processing based on the perceived location or approaching motion of a stimulus, contribute to the definition of peripersonal space itself as well as of its boundary. Predictions, and then in turn peripersonal space representations, are driven by priors created by repeated and consistent associations between exteroceptive signals in the space surrounding the body and somatosensory events on the body. Showing any evidence of a rudimental representation of peripersonal space in very young infants would pave the way to an important discussion on whether the multisensory associations characterizing peripersonal space could perhaps be considered to a certain extent innate or rather whether they develop during prenatal life, thanks to multisensory experiences *in utero*.

The present set of studies aimed at extending the line of research investigating adults’ peripersonal space boundaries to the earliest stages of life, exploring whether peripersonal space could be considered as a delimited portion of space with identifiable boundaries already soon after birth. To do so, we adapted the audio-tactile interaction task initially developed by Canzoneri *et al*.^37^ in order to be able to use it with newborns, hypothesizing that, if newborns have a rudimentary representation of peripersonal space as a delimited portion of space, their pattern of responses will resemble that showed by adults.

A first pilot study showed a clear modulation of newborns’ saccadic reaction times (sRTs) to a peripheral visual target appearing on the screen immediately after the termination of an audio-tactile stimulation in which the auditory stimulus was presented at three different intensities. In particular, newborns’ sRTs did not simply decrease continuously as a function of the perceived intensity of the sound: on the contrary, sRTs were significantly faster when the auditory stimulus was perceived at the intermediate intensity (Sound 3), compared to the softer one (Sound 1), while they were similar between the intermediate intensity and the louder one (Sound 5). This pattern of sRTs closely resembled the pattern of RTs showed by adults in Canzoneri *et al*.’ study^37^. In light of the results of the pilot study, we decided to further explore newborns’ responses in this task, in particular investigating their sRTs when the auditory stimuli were presented at other intensity levels. Importantly, we also wanted to rule out the possibility that newborns were responding solely to the auditory stimulus, ignoring the tactile stimulation. In fact, it might be suggested that louder sounds could on their own modulate newborns’ alertness state, irrespective of any supposed peripersonal spatial representation. To control for this, in Study 1 we included a second group of participants who experienced only the auditory stimulation, but not the tactile one. The results of Study 1 showed that newborns sRTs were modulated by the intensity of the sound when they were presented with audio-tactile stimuli, but not when they were presented with auditory stimuli only. This confirmed that the effect of the intensity of the sound on newborns’ sRTs to a peripheral visual target was indeed due to the combination of simultaneous auditory and tactile stimulation and was not simply function of the intensity of the sound on its own. In particular, in the Audio-tactile stimulation group the sRTs decreased significantly between Sounds 1 and 2, while they were not significantly different from each other between Sounds 0 and 1, nor between Sounds 2, 3 and 5. These results suggest that newborns’ reaction times to a peripheral visual target presented immediately after an audio-tactile stimulation are significantly speeded up when the auditory stimulus is louder than a certain critical intensity (between Sounds 1 and 2).

It is important to remark that, given the specific population taking part to our studies, we necessarily needed to adjust the task used, making several changes. For example, we decided to measure saccadic reaction times to a peripheral visual target presented to the newborns after the audio-tactile (or just the auditory) stimulation. Clearly, any of these changes could have significantly mined the ability of the task to measure peripersonal space. However, the presented results suggest that the this is not the case and that, on the contrary, the modality of collection of the reaction times does not seem to influence the changes in their speed once an external cue is perceived within peripersonal space. This suggestion is supported by the findings of several studies^4,46,47^ that despite focusing around different body parts and despite using different tasks, all seem to have been able to measure index peripersonal space and identify its boundaries.

In Canzoneri *et al*.’s study^37^, the authors asked the participants to judge the distance of the sound source at each of the delays when the tactile stimulus was perceived. This allowed them to conclude that the critical intensity of the moving sound in response to which the reaction times decreased significantly did indeed correspond to a specific location in space. We could not find a suitable way to investigate whether newborns perceived static sounds of different intensities as located at different distances from the body. However, given that intensity is the sound feature that better specifies sound location in depth^42^, we speculate that sounds with different intensities could be perceived at different distances from the body in newborns prior to perceptual narrowing^48^. In turn, this speculation leads us to suggest that the sound intensity in which correspondence saccadic reaction times decreased significantly could be considered, drawing a parallel with what suggested by Canzoneri *et al*.^37^, as the boundary of a rudimentary representation of peripersonal space in newborns. This suggests that already soon after birth peripersonal space may be represented as a delimited portion of space where it is easier for newborns to match multisensory stimuli taking place simultaneously and that it may be possible to determine its boundaries.

In our studies, we demonstrated a spatially delimited facilitation of multisensory matching in newborns infants. This is particularly interesting within the theoretical reflection of peripersonal space in light of the most recent conceptual developments concerning it. Recently, researchers widely highlighted the important and intrinsic links between peripersonal space and prediction of incoming interactions between entities in space and the body^3,35,36^. In fact, it has been suggested that predictive mechanisms that anticipate an impending contact based on the perceived location or approaching motion of an object in space could drive the facilitation of tactile processing that takes place when an exteroceptive stimulus is perceived near the body and defines peripersonal space and its boundaries. The predictions that we make on the likelihood of an incoming tactile sensation on our body based on exteroceptive cues signalling the position or trajectory of an object in space are driven by priors built through previous experience of the existing relationship between these multisensory perceptual aspects. Given the extremely limited exposure to multisensory events involving an object in peripersonal space and a subsequent touch on the body that newborn infants may have experienced in the first few days of their lives, the findings reported here necessarily lead to the question of when and how these associations formed in development. Two main lines of speculation may be suggested. On one hand, it might be speculated that the ability to make a predictive association between an event signalled by visual or auditory cues taking place near the body with a subsequent tactile event signalled by somatosensory cues happening on the body, is to a certain extent innate, provided to the developing infant through phylogenetical development. On the other hand, it may be suggested that soon after birth newborns might be readily capable of making these associations, and therefore may show a facilitation of multisensory matching when events happen within peripersonal space, thanks to their prenatal multisensory experiences. In particular it can be speculated that already in the womb foetuses could experience the link between nearby stimuli, auditory in particular, and temporally and spatially related tactile stimuli perceived through the uterine wall. Using 4D ultrasounds to investigate foetal orienting and heart rate responses^49^ to tactile stimuli presented through the uterine wall in close temporal and spatial proximity would be a viable way to tackle this theoretically fundamental question and explore whether prenatal experience could be the main factor driving the early associative ability highlighted by our findings. A second important aspect to further investigate would be the quality and role of the early associations shown by newborn infants. It would in fact be important to disentangle whether these could be already considered as inferential associations between an event in peripersonal space and its bodily consequences or whether newborns’ enhanced multisensory matching of auditory and tactile events taking place near the body could be functional to driving their attention to these same associations, increasing their chance to experience them and, in this way, build the predictive link underlying the adult conception of peripersonal space.

Following up on these findings, it would also be important to investigate if and how the dimensions of peripersonal space change through the lifespan. In fact, at this stage we can only speculate about this, as we do not know how far in space the auditory stimuli were perceived by newborn participants, nor whether the absolute positions in space where a sound with the same intensity is perceived by adults and newborns are at all similar, nor even if differences in sound intensity are indeed sufficient to newborns to perceive sound sources at different distances from the body. In their study, Canzoneri *et al*.^37^ ran a sound localization task with 7 naïve participants in order to demonstrate that the auditory sound position was actually perceived at different locations in space at each of the different time delays when the tactile stimulus was presented. However, we could not yet find a way to adapt this task in order to use it with newborns. A viable way of investigating the variations in peripersonal space dimensions between very young infants and adults would be to replicate the study with a group of adults, using static vs dynamic sounds. This would allow us first of all to investigate whether the critical distance after which adults’ reaction times are speeded up would be the same as that found by Canzoneri *et al*.^37^, irrespective of the differences between the two tasks. Secondly, and most importantly, identifying the critical distance after which adults’ RTs are speeded up using this task would allow us to directly compare the distance of the boundary of peripersonal space from the body in adults and any developmental population. Provided that the distance of the peripersonal space boundary in adults measured with this task would be the same as that measured by Canzoneri *et al*.^37^ (i.e. the distance from the body of an auditory stimulus whose intensity is 62.5 dB), newborns’ peripersonal space as measured in this set of studies might be considered slightly bigger than adults’ peripersonal space. In fact, sRTs in newborns were first significantly speeded up when the sound intensity was 59 dB, hence when the sound source was supposedly perceived as further away from the participants’ body than it was in the adult sample. We can speculate that this might be due to the fact that newborns need positive interactions with other humans (caregivers in particular) to guarantee their survival and therefore they may need a larger peripersonal space, capable of including also others within it. In fact, it has been demonstrated that peripersonal space boundaries are sensible to the presence of others in the far space and are shaped by the quality of the interaction with them, in particular expanding to include another person and his/her peripersonal space after a cooperative interaction^38^.

Irrespective of any speculations on the comparison between newborns and adults’ peripersonal space, the findings of the present set of studies show that newborns with minimal postnatal experience show spatially delimited facilitation of multisensory interactions taking place in the space near the body. This suggests that young infants could already show a rudimentary representation of peripersonal space as a bounded portion of space, which we may speculate being already invested of a special importance soon after birth, as the portion of space where newborns’ earliest significant physical and social interactions with the environment will take place. In light of peripersonal space intrinsic links with prediction of upcoming events involving the body, the presence of these early rudimentary associations between exteroceptive signals and somatosensory cues highlights the need for further investigation and theoretical discussion on the developmental origins of these same associations.

## Method

### Participants

The final sample of the pilot study included 8 newborns (5 female) aged between 16.5 and 75 hours when they took part. Four additional newborns participated but were excluded due to an experimental error (n = 1) or because they did not complete enough trials (n = 3). All the participating newborns met the screening criteria of normal delivery, birth weight >2500 g, gestational age >37 weeks and Apgar index score between 8 and 10 at the fifth minute of life. No abnormalities were present at birth. The 8 newborns in the final sample had a mean age of 40.22 hours (*SD* = 20.16) at test, a mean birth weight of 3436.25 g (*SD* = 432.27) and a mean gestational age of 39.48 weeks (*SD* = 1.01).

The final sample of Study 1 included 31 newborns aged between 12 and 94 hours at the time of testing. Seventeen additional newborns participated but were excluded due to an experimental error (n = 1), sleepiness (n = 4), because they did not complete enough trials (n = 11) or because of a suspect hearing problem (n = 1). All the participating newborns met the screening criteria of normal delivery, birth weight >2500 g, gestational age >37 weeks and Apgar index score between 8 and 10 at the fifth minute of life. No abnormalities were present at birth. The newborns who took part in Study 1 were randomly divided in two groups: one group experienced audio-tactile stimulation (“Audio-tactile stimulation group”, N = 16, 8 female), while the other experienced unisensory, auditory only stimulation (“Auditory stimulation group”, N = 15, 9 female). The newborns in the Audio-tactile stimulation group had a mean age of 64.98 hours (*SD* = 15.65) at test, a mean birth weight of 3435.63 g (*SD* = 328.17) and a mean gestational age of 40.14 weeks (*SD* = 1.34); the newborns in the Auditory stimulation group had a mean age of 40.16 hours (*SD* = 20.10) at test, a mean birth weight of 3397.33 g (*SD *= 384.88) and a mean gestational age of 40.21 weeks (*SD* = 1.36).

Testing took place when newborns were awake and alert, usually immediately before feeding. The parents were informed about the procedure and provided written informed consent to their child’s participation. The study protocol was designed in accordance with the relevant regulations and approved by the Ethical Committee of Psychology Research (University of Padova).

### Apparatus

The studies were conducted at the Paediatric Unit of the Hospital of Monfalcone (GO, Italy), where the newborns were born. During the studies, the newborns sat on the experimenter’s lap and watched the stimuli presented on a monitor (24″) in front of them. Newborns’ eye level was aligned to the centre of the screen and the distance between their face and the monitor was about 35 cm, to maximize their visual acuity^50,51^. Black cardboard and black curtains covered the area around the monitor to prevent external stimuli from engaging the newborns’ attention. The auditory stimuli were conveyed from two loudspeakers positioned one under the left and one under the right halves of the monitor, while the tactile stimuli (gentle paintbrush strokes) were applied by hand by one of the experimenters. A video camera located on top of the screen recorded the newborns’ eyes allowing subsequent offline coding of their eye movements. An additional small screen, placed outside the newborns’ view, allowed the experimenter to monitor their head position throughout the experiment. The experimenter holding the newborns was always unaware of the ongoing trial and was instructed to constantly focus on the monitor showing the newborns’ mirrored head position, therefore being unable to see the stimuli. Stimuli were presented using E-Prime 2.0.10.

### Stimuli and experimental procedure

We adapted the audio-tactile interaction task developed by Canzoneri *et al*.^37^ in order to use it with newborns. In the original task, the participants were presented with a sample of pink noise of raising or falling intensity. During the presentation of the auditory stimulus, they felt a touch on their hand, which happened at five different delays from the onset of the auditory stimulus, hence occurring when the sound source was perceived at five different distances from the hand. The participants were asked to verbally respond to the touch as rapidly as possible, trying to ignore the sound. In our adaptation, we decided to: i) measure newborns’ saccadic latency to two visual targets appearing on the screen immediately after the audio-tactile stimulation terminated (sRTs), ii) use static – rather than dynamic – auditory stimuli, that were perceived at different distances from the body, iii) use samples of sinusoidal waveforms instead of samples of pink noise, and iv) deliver the tactile stimulation gently stroking the newborns’ forehead using a paintbrush, to avoid priming the saccadic responses to either side of the screen with a lateralized touch.

Auditory stimuli characterized by different intensities were presented, as intensity is the feature that better accounts for sound location in depth^42^. To choose the intensity of the auditory stimuli, we calculated the intensity of the sound at each of the time delays when the tactile stimulation was delivered in Canzoneri *et al*.^37^: the sample of pink noise used changed from 55 to 70 dB and lasted 3000 ms, with an intensity change of 0.005 dB each ms (Sound onset = 55 dB, T1 = 56.5 dB, T2 = 59 dB; T3 = 62.5 dB; T4 = 76 dB; T5 = 68.5 dB, Sound offset = 70 dB). In the pilot study, we decided to use three sounds whose intensities corresponded to those at sound onset, sound offset and T3 in Canzoneri *et al*.^37^. In light of the results of the pilot study, in Study 1 we added one auditory stimulus whose intensity corresponded to that at T2 in Canzoneri *et al*.^37^ and another one whose intensity was softer than the intensity at sound onset (47 dB). Each study comprised as many Sound Intensity conditions as the number of auditory stimuli used. Table 4 summarises the sound intensity conditions included in each study and their correspondence with the sound intensity at each of the touch delivery delays in Canzoneri *et al*.^37^.

**Table 4:** The table lists the intensity of the auditory stimuli in each Sound Intensity condition of the Pilot Study and Study 1 and their correspondence with each of the touch delivery delays in Canzoneri *et al*.^37^.

	Sound 0	Sound 1	Sound 2	Sound 3	Sound 5
Pilot Study	—	55 dB	—	62.5 dB	70 dB
Study 1	47 dB	55 dB	59 dB	62.5 dB	70 dB
Correspondence with Canzoneri *et al*.^37^	—	Sound onset	T2	T3	Sound offset

**Figure 3:**
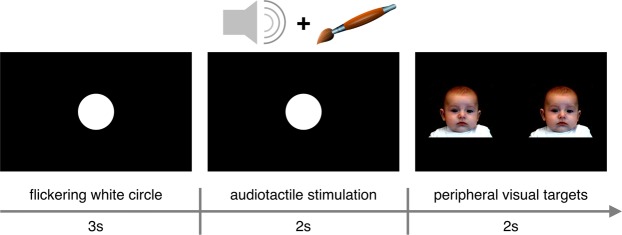
Experimental procedure. The newborns were presented with a flickering white circle on a black background for 3 s; then, one of the three (pilot study) or five (Study 1) possible auditory stimuli was presented for 2 s. At the same time, the newborns’ forehead was gently and slowly stroked (only once) with a paintbrush. In the meantime, the white circle kept flickering in order to keep the newborns’ attention focused in the centre of the screen. Finally, two peripheral targets appeared and remained on the screen for another 2 s, before the next trial started.

To attract newborns’ attention to the centre of the screen, we presented a white circle flickering at a frequency of 2.5 Hz in the middle of a black background. This was necessary to maintain the newborns awake and alert during the study and to maintain their fixation point to the centre of the screen, maximizing the chances of obtaining valid responses. The attention getter was presented alone for 3000 ms, then the auditory stimulus was introduced, for further 2000 ms. The auditory stimuli were samples of a sinusoidal waveform of constant frequency (8000 Hz), played at the already specified intensities. The intensity of the auditory stimuli was measured in the position where the newborns’ head was during testing, at the average room conditions (environmental noise, lighting and set up). While the auditory stimulus was presented, one of the experimenters applied the tactile stimulation by hand, gently stroking the newborns’ forehead with a paintbrush. During the audio-tactile stimulation, the white circle kept flickering on the screen, in order to keep the newborns’ attention in the same position and avoid eye movements. As soon as the audio-tactile stimulation terminated, two visual target stimuli appeared on peripheral sides of the screen and remained visible for 2000 ms. As it is customary in infancy research, we presented the stimuli in the peripheral sides of the screen, based on infants’ subcortically driven tendency to readily orient towards stimuli in the temporal visual hemifield^41^. The two peripheral targets were two identical infant faces (the parent of the portrayed infant provided consent to online open-access publication) on a black background, whose pupils were 1 cm in diameter so that they could be seen by newborns^52^. As soon as the visual targets disappeared, a new trial started, following the same sequence (Fig. 3). In the pilot study, the newborns were presented with a maximum of 30 trials (10 per condition) in random order, as long as their attention lasted; in Study 1, instead, up to 31 trials were presented (to keep the total experiment length similar to that of the pilot study), 7 each for Sound Intensity conditions 1, 3 and 5 and 5 each for the newly introduced Sound Intensity conditions 0 and 2.

### Analyses

The newborns’ eye movements were recorded throughout the experimental session and later coded offline by an expert observer, blind to the Sound Intensity condition of each trial, who recorded newborns’ sRTs, i.e., the “latency of the first eye movement away from the centre towards the peripheral target” (^53^, p. 176). The trials were considered valid only if the newborns were looking at the centre of the screen immediately before the presentation of the peripheral targets. In the pilot study, the newborns were included in the final sample only if they completed at least two valid trials per Sound Intensity condition, while in Study 1 they were included if they had completed at least two valid trials per at least 4 out of the 5 Sound Intensity conditions. A second observer recorded the sRTs for the 10% of the participants (n = 4); the average intraclass correlation coefficient (absolute agreement) between the two raters was ICC_(2,2)_ = 0.903. Analyses were run using R software^54^ and the R “lme4” package^55^ for mixed effects models.

## Data Availability

The datasets generated during and/or analysed during the current study are available from the corresponding author.
